# Modulation of Huntingtin Toxicity by BAG1 is Dependent on an Intact BAG Domain

**DOI:** 10.3390/molecules15106678

**Published:** 2010-09-28

**Authors:** Jan Liman, Kamila Sroka, Christoph P. Dohm, Sebastian Deeg, Mathias Bähr, Pawel Kermer

**Affiliations:** 1 Deptment of Neurology, University of Göttingen, Robert-Koch Str. 40 37075 Göttingen, Germany; 2 DFG-Research Center for Molecular Physiology of the Brain (CMPB), Humboldtallee 23, 37075 Göttingen, Germany; E-Mails: jliman@gwdg.de (J.L.); cdohm@gwdg.de (C.P.D.); mbaehr@gwdg.de (M.B.); 3 Merz Pharmaceuticals, R&D CNS, In vitro Pharmacology, Eckenheimer Landstrasse 100, 60318 Frankfurt, Germany, E-Mail: kamilasroka@yahoo.co.uk (K.S.)

**Keywords:** BAG1, Huntington’s disease, Chaperone system, Siah1

## Abstract

Huntington´s disease, one of the so-called poly-glutamine diseases, is a dominantly inherited movement disorder characterized by formation of cytosolic and nuclear inclusion bodies and progressive neurodegeneration. Recently, we have shown that Bcl-2-associated athanogene-1 (BAG1), a multifunctional co-chaperone, modulates toxicity, aggregation, degradation and subcellular distribution *in vitro* and *in vivo* of the disease-specific mutant huntingtin protein. Aiming at future small molecule-based therapeutical approaches, we further analysed structural demands for these effects employing the C-terminal deletion mutant BAGΔC. We show that disruption of the BAG domain known to eliminate intracellular heat shock protein 70 (Hsp70) binding and activation also precludes binding of Siah-1 thereby leaving nuclear huntingtin translocation unaffected. At the same time BAGΔC fails to induce increased proteasomal huntingtin turnover and does not inhibit intracellular huntingtin aggregation, a pre-requisite necessary for prevention of huntingtin toxicity.

## 1. Introduction

Bcl-2 associated athenogene 1 (BAG1) is an antiapoptotic co-chaperone of Hsp70 and its cognate protein Hsc70 (hereafter referred to as Hsp70) [1]. It binds to the ATPase domain of Hsp70 through its C-terminus. Via its N-terminal ubiquitin-like motif it was found to bind to the E3 ligase CHIP (carboxyl terminus of Hsc70-interacting protein) and to the α7-subunit of the proteasome [2,3]. Thus it is thought, that unfolded Hsp70 substrates are delivered by BAG1 to the proteasome for degradation. BAG1 can also bind to Raf1 kinase, thereby modulating cell growth and differentiation via the ERK pathway [4,5]. This binding competes with its binding to Hsp70, implying that BAG1 might act as a switch between cellular stress, growth and differentiation. In neurons, BAG1 has been shown to promote differentiation [6]; its importance for the neuronal system is underlined by the fact, that mice lacking BAG1 are lethal *in utero* due to massive apoptosis in the nervous system, heart and liver [7]. On the other hand, BAG1 overexpression can protect from ischemic cell death and plays an important role in axonal regeneration *in vivo* [5,8].

Another binding partner for BAG1 is Siah1, a p53 inducible pro-apoptotic protein and E3 ligase [9] linking BAG1 to the pathogenesis of Huntington’s disease. Huntington’s disease (HD) is a dominantly inherited fatal neurodegenerative disease resulting from a glutamine-encoding CAG trinucleotide repeat expansion in exon 1 of the *IT15* gene coding for huntingtin (htt) [10]. It is ubiquitiniously expressed, but the mutation primarily affects striatal and cortical neurons. The pathomechanisms of HD still remain unclear. It is thought that gene transcription [11,12,13,14], cellular transport [15,16], mitochondrial dysfunction, as well as impairment of the ubiquitin-proteasome system [17,18,19] play a critical role in the disease. One important feature of htt is the cleavage of the N-terminal glutamine-expansion containing fragment. In this regard, cleaved mutated htt enters the nucleus in complex together with GAPDH and Siah1 [20]. This translocation to the nucleus is believed to be a major step in the pathogenesis of HD [21,22]. In a previous study, we indeed were able to show, that BAG1 reduces aggregation and accelerates degradation of mutant htt in a proteasome- and Hsp70-dependent manner. Moreover, it reduces nuclear levels of mutant htt. Interestingly BAG1 downregulates cellular levels of Siah1 and overexpression of Siah-1 abolishes BAG1 effects [23].

Aiming for possible therapeutic strategies we further analysed the structural demands of BAG1. Therefore, we employed a C-terminal deletion mutant of BAG1 disrupting the functional BAG domain, through which binding to Hsp70 and Siah1 is provided.

## 2. Results and Discussion

### 2.1. BAGΔC is not binding and not able to downregulate Siah1

**Figure 1:**
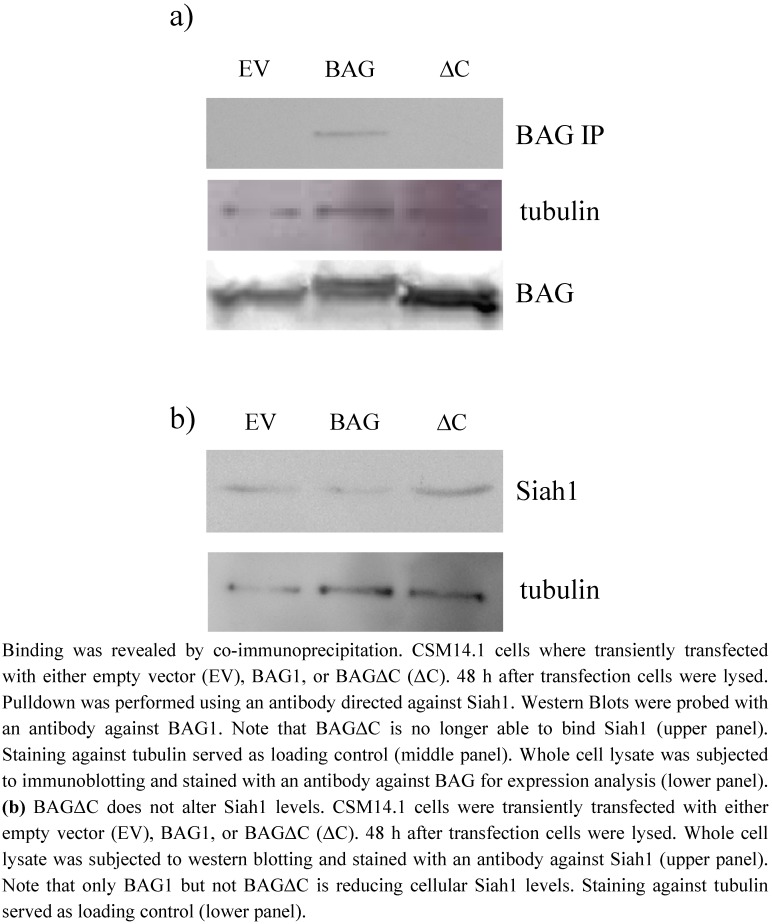
(**a**) BAG1, but not BAGΔC binds to SIAH.

We transiently expressed BAG1 and BAGΔC in CSM14.1 cells as shown before. By co-immunopreciptitation experiments we confirmed binding of full length BAG1 to Siah1 (Figure 1a) [23] while binding of the C-terminal deletion mutant of BAG (BAGΔC) to Siah1 is not detectable. (Figure1a). As we and others could show previously, that BAG1 is inhibiting or down-regulating Siah1 *in vitro*, we were interested if BAGΔC could still influence Siah1 levels in cells [9,23]. As expected, we observed similar Siah1 levels in cells overexpressing BAGΔC when compared to controls, while Siah1 levels in presence of BAG1 were reduced (Figure 1b). 

### 2.2. Reduction of htt inclusions

**Figure 2:**
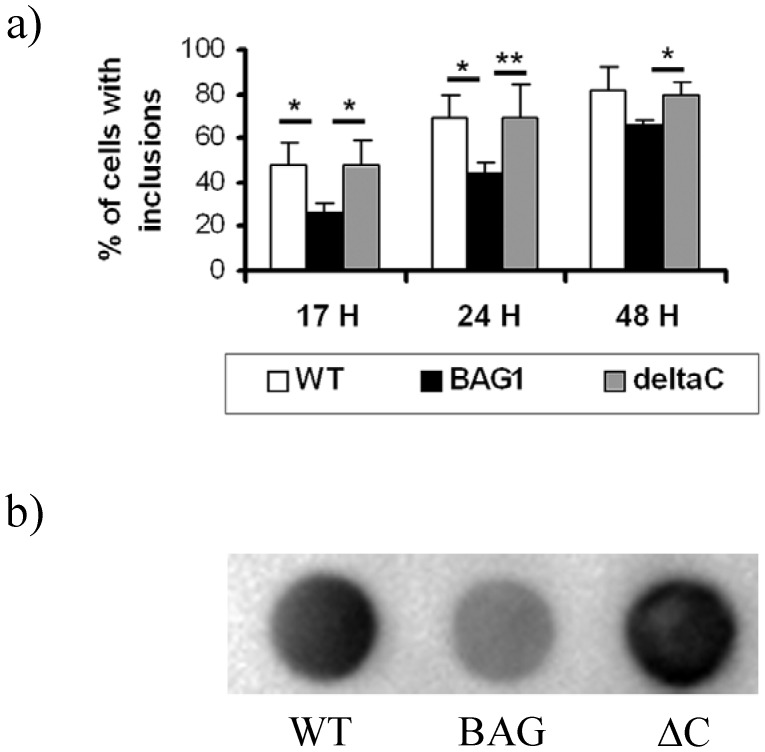
BAG1, but not BAGΔC decreases mut-htt aggregation.

Previous results suggested that BAGΔC acts in a dominant negative way on protein refolding [24]. Thus, we hypothesized that the C-terminal deletion mutant of BAG1 would not be able to reduce htt aggregate formation. To test our hypothesis, we first employed a C-terminal eGFP fused htt protein transfected to CMS14.1 cells [23]. As known from our previous study, BAG1 colocalizes to inclusion bodies and is able to reduce the amount of aggregates significantly. We then co-transfected the eGFP-htt together with BAGΔC and indeed could not find any significant reduction of inclusion bodies compared to controls (Figure 2a). To validate this data, we employed a filter retardation assay established before [25] and observed a significant reduction of htt aggregates in cells transfected with BAG1, an effect abolished by disruption of the BAG domain in cells over-expressing BAGΔC (Figure2b). 

CSM 14.1 wt, BAG1 and BAGΔC (ΔC) cells were transfected with htt-mut fused to eGFP. The percentage of cells that contained inclusions quantified. Cells stably expressing BAG1 are less likely to develop inclusions than wild type cells or cells stably expressing BAGΔC at all time points investigated post-transfection (a). Reduction in aggregate formation can also be observed in a filter retardation assay (b), where captured aggregates were detected with an antibody directed against the polyQ stretch. At least 400 cells were scored for each condition. The data represent mean values and SEM of three independent experiments (Student’s *t*-test, *, p < 0.05, **, p < 0.01, a; ANOVA,*, p < 0.05, b).

### 2.3. BAGΔC is no longer able to enhance htt degradation

**Figure 3:**
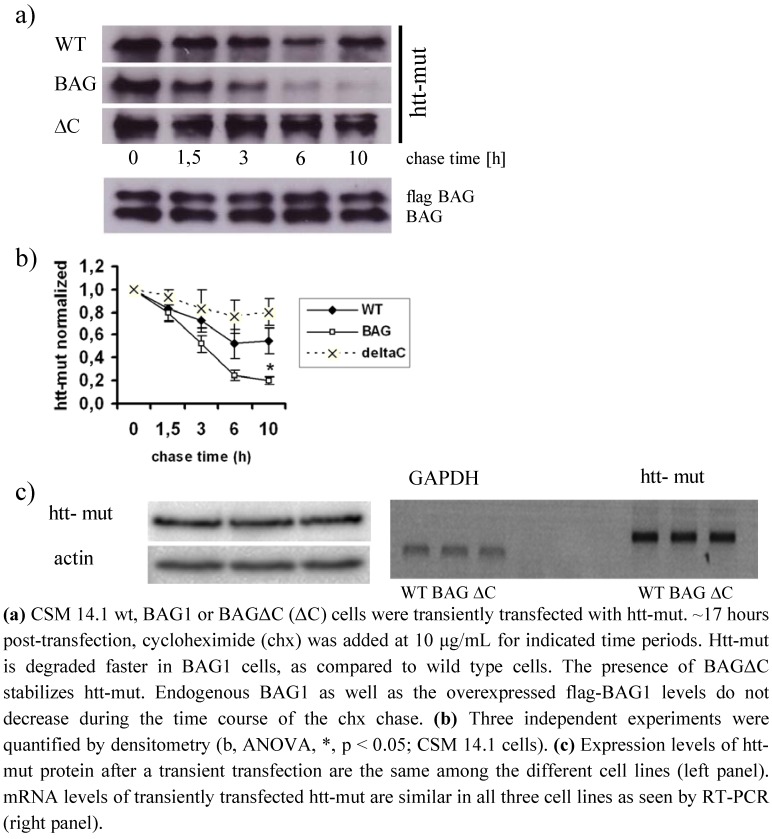
BAGΔC does not enhance htt-mut degradation.

We recently were able to show that BAG1 reduces htt aggregate formation by increasing htt turnover in a proteasome dependent manner, as BAG1 is able to interact with the α7-subunit of the proteasomal core [23]. To investigate whether BAGΔC is still able to influence htt-mut degradation, we performed a cycloheximide chase experiment. In contrast to BAG1, BAGΔC did not increase mut-htt turnover (Figure 3a). In cells stably expressing BAG1 htt-mut is cleared faster with a half-life of ~3.3 hours as compared to wild type cells in which it had a half-life of ~4.9 hours (Figure 3b). In line with previous results from our laboratory where BAGΔC appeared to have a dominant negative effect on protein folding, mutant htt seemed to be even more stable in the presence of BAGΔC (half-life ~9,5 hours) than in wild type cells. (Figure 3b) [24]. Expression levels of htt remained unaltered by the presence of BAG1 or BAGΔC (Figure 3c).

### 2.4. htt translocation to the nucleus *is not modulated by BAGΔC*

**Figure 4:**
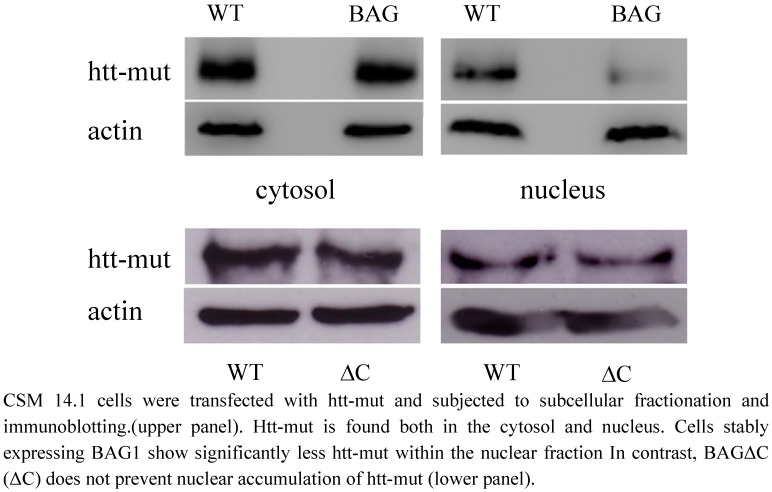
BAG1 influences htt-mut subcellular localization.

Mutant htt was found both in the cytoplasm and in the nucleus when overexpressed in CSM14.1 cells. This is also in accordance with previous studies showing nuclear accumulation leading to toxicity of the mutant protein [21,22]. In cells stably expressing BAG1, nuclear htt-mut levels were decreased compared to wild type cells (Figure 4 upper right panel) In contrast, over expressing BAGΔC fails to reduce the amount of htt-mut inside the nucleus (Figure 4 lower right panel).

## 3. Experimental

### 3.1. Protein extract preparation

Cells were washed, scraped in ice-cold PBS and pelleted. Cell lysates were prepared using a buffer containing 50 mM TRIS-HCl (pH 7,4), 150 mM NaCl, 1% Triton-X 100 and complete protease inhibitor cocktail (Roche) for 15 minutes at 4°C, followed by 10 minutes centrifugation at 13,000 rpm at 4 °C. The pellets which were further used for the filter retardation assay were resuspended in the lysis buffer and sonicated.

### 3.2. Filter retardation assay

For the filter retardation assay protein extracts were heated at 98 °C for 3 min in 2% SDS and 50 mM DTT and filtered through a 0.2 µm cellulose acetate membrane (Schleicher & Schuell, Dassel, Germany) using a dot-blot filtration unit. The membranes were further processed for immunodetection.

### 3.3. Antibodies

The following antibodies were used at corresponding dilutions: rabbit polyclonal CAG53b, 1:2,000 (a gift from E.E, Wanker), mouse monoclonal 1C2, 1:1,000 (Chemicon), rabbit polyclonal BAG 1680 [6], 1:1,000, goat polyclonal Siah1, 1:1,000 (Everest Biotech), mouse monoclonal b-tubulin, 1:1,000 (Sigma), actin, 1:5,000, (Chemicon).

### 3.4. Immunoblotting

For Western blotting, a Mini Trans-Blot Cell setup (7.5 × 10 cm blotting area, Bio-Rad) was used. After the blotting step, the membrane was incubated in blocking solution (5% milk in TBS-T) for 1 hour at room temperature to avoid unspecific binding of the antibody, at 4 °C overnight and with secondary HRP-conjugated antibodies for 1 h at room temperature. The membrane was washed 3 x 20 minutes with TBS-T after each antibody incubation. To develop membranes ECL was used and applied for 2 minutes onto the membrane. Films (Amersham, Hyperfilm ECL) were exposed to the membrane and developed using AGFA Curix 60 table-top processor.

### 3.5. Co-immunoprecipitation

Cell lysates were prepared as described above. Affinity beads (Sigma) were washed twice with lysis buffer and 25 μL beads were added to each tube. The beads were then incubated with cellular lysates for 2 hours at 4 °C with rotation. Subsequently the pulldown anitibody directed against Siah1 was added. Lysates were again incubated at 4 °C overnight. Next they were spun down, washed twice with the lysis buffer and twice with TBS buffer (400 μL, 5 minutes each washing step). Proteins bound to the beads were eluted by adding 20 μL 2X SDS sample buffer for 5 minutes at 95 °C.

### 3.6. Cell culture

CSM 14.1 wt cells were grown in DMEM supplemented with 10% FCS and penicillin/streptomycin in 32 °C. Stably transfected CSM-BAG1 and CSM-ΔC were maintained in the same medium with an addition of puromycin. HEK293T cells were grown in DMEM supplemented with 10% FCS and penicillin/streptomycin in 37 °C. For transient transfections Lipofectamine 2000 (Invitrogen) was used. 

### 3.7. Transient transfections

Most transfections were done using Lipofectamine 2000 reagent. In case when large amount of cells needed to be transfected, a more cost-efficient calcium phosphate method was used. Lipofectamine transfections were done according to the manufacturers provided protocol.

### 3.8. Calcium phosphate transfection

Cells were plated on a 10 cm dish, 45 μg DNA was mixed with 125 μL of 2 M CaCl2 and H2O to achieve a total volume of 1 mL. One mL of 2× HEPES buffer was added, mixed and incubated for 15 minutes at room temperature. During the incubation time, fresh medium was added to the cells. Following the incubation time, the mixture was added dropwise to the cells. Twelve hours post transfection cell were washed in PBS and fresh medium was added. Gene expression was assayed 36 hours later.

### 3.9. Densitometry analysis

To quantify Western blot protein bands, ImageJ software was used. The blot images were opened in the program as tiff files. A same size rectangular selection was drawn around each band and lanes were selected using Analyze > Gels > Select Lane function. Histograms representing the intensity of pixels in the selected areas were created using Analyze > Gels > Plot Lanes function. The area under the histograms, representing pixel intensity in the selected area, was limited using the straight line selection in order to integrate the signal. The values of the histogram area were obtained by clicking inside the histogram with the wand tracing tool. Values corresponding to the protein of interest were normalized by dividing them by the values of the loading control to account for differences in the amount of protein loaded.

## 4. Conclusions

BAG1 is a multifunctional protein involved in central nervous system development and regeneration. It is believed to act as a switch between the proteasomal and chaperone mediated protein detoxification and is involved in cell cycle and regeneration. It was shown previously by us and others that BAG1 can ameliorate htt toxicity *in vitro* and *in vivo* [23,26]. These results suggested this effect to be due to increased proteasomal degradation with BAG1 acting as an accelerator of Hsp70 mediated protein refolding on the one and proteasomal degradation on the other hand [23,24]. Furthermore, we could show that BAG1 also downregulates Siah1 levels in cells thereby preventing the transfer of mutated htt into the nucleus, which is believed to be another key event in htt toxicity [20,22,23]. In our current study, we tried to gain more insight into the different functional parts of BAG1 and their potential therapeutical role. We therefore applied BAGΔC, a C-terminal deletion mutant of BAG1 lacking the BAG domain, which is essential. for Hsp70 binding and activation [24].We show here that this mutant is also not able to bind Siah1 anymore (Figure 1a) [9]. Indeed BAGΔC also fails to downregulate Siah1 levels *in vitro* when compared to full length BAG1 (Figure 1b). Consequently, nuclear htt translocation is not reduced (Figure 4). This supports our previous finding that downregulation of Siah1 by BAG1 is due to a direct interaction and probably dependent on increased proteasomal degradation of Siah1, as blockage of the proteasomal system can reverse this effect [23]. 

Another hallmark of BAG1 is its ability to prevent mutated htt aggregation in a proteasome dependent manner *in vitro* [23]. This bears up the theory that BAG1 acts as a “shuttle” between the chaperone system and the proteasomal system for more rapid degradation of misfolded proteins [3]. In line with this, BAGΔC is not able to provide aggregate reduction of mutated htt (Figure 2) suggesting BAG1 as HSP70 “assistant” in binding and refolding mutated htt before shuttling it to the proteasomal system where BAG1 can accelerate protein degradation.
